# NK cells associate with ALS in a sex- and age-dependent manner

**DOI:** 10.1172/jci.insight.147129

**Published:** 2021-06-08

**Authors:** Benjamin J. Murdock, Joshua P. Famie, Caroline E. Piecuch, Kristen D. Pawlowski, Faye E. Mendelson, Cole H. Pieroni, Sebastian D. Iniguez, Lili Zhao, Stephen A. Goutman, Eva L. Feldman

**Affiliations:** 1 Department of Neurology and; 2 Department of Biostatistics, School of Public Health, University of Michigan, Ann Arbor, Michigan, USA.

**Keywords:** Immunology, Neuroscience, ALS, NK cells

## Abstract

NK cells are innate immune cells implicated in ALS; whether NK cells impact ALS in a sex- and age-specific manner was investigated. Herein, NK cells were depleted in male and female SOD1^G93A^ ALS mice, survival and neuroinflammation were assessed, and data were stratified by sex. NK cell depletion extended survival in female but not male ALS mice with sex-specific effects on spinal cord microglia. In humans, NK cell numbers, NK cell subpopulations, and NK cell surface markers were examined in prospectively blood collected from subjects with ALS and control subjects; longitudinal changes in these metrics were correlated to revised ALS functional rating scale (ALSFRS-R) slope and stratified by sex and age. Expression of NK cell trafficking and cytotoxicity markers was elevated in subjects with ALS, and changes in CXCR3^+^ NK cells and 7 trafficking and cytotoxicity markers (CD11a, CD11b, CD38, CX3CR1, NKG2D, NKp30, NKp46) correlated with disease progression. Age affected the associations between ALSFRS-R and markers NKG2D and NKp46, whereas sex impacted the NKp30 association. Collectively, these findings suggest that NK cells contribute to ALS progression in a sex- and age-specific manner and demonstrate that age and sex are critical variables when designing and assessing ALS immunotherapy.

## Introduction

ALS is a lethal neurodegenerative disease characterized by progressive cortical and spinal motor neuron death, resulting in loss of voluntary motor control (1). Subjects experience progressive muscle weakness, atrophy, paralysis, and eventually death, and mean survival is only 2–4 years from diagnosis. Despite the devastating nature of ALS, treatment options are still severely limited and new treatment options are needed.

Previous studies demonstrate that the immune system contributes to ALS progression in both humans and in mouse models (2, 3). The innate immune system in particular — which includes multiple cell types, cytokines, and complement that react rapidly to general, nonspecific threats in the body — is implicated in disease progression (4–7) and may be a viable therapeutic target in ALS. As part of the innate immune system, NK cells not only protect the body by destroying the body’s own cells when they become infected or cancerous (8) but also eliminate previously damaged cells, including those in the nervous system (9). Healthy, uninfected cells, including motor neurons, are normally protected from NK cell damage by expression of major histocompatibility complex I in mice, or human leukocyte antigen A-C surface markers in humans; these protective proteins are lost on spinal cord motor neurons in both ALS mice and humans with ALS rendering them vulnerable to NK cell damage (10). Moreover, NK cells are elevated in peripheral blood of subjects with ALS (4, 11), infiltrate the CNS of ALS mice and human subjects (12, 13), and drive proinflammatory microglial activity (13). These findings suggest that NK cells play a role in ALS and may be viable therapeutic targets in ALS.

The potential effect of NK cells in ALS, and the immune system as a whole, may be complicated by both age and sex because both can influence immune responses in healthy individuals (14) and during various disease states (15). Immune-based sex differences can also affect neurodegeneration: loss of microglia microRNA expression exacerbates tau pathology in male PS19 Alzheimer’s mice (16); and in humans, peripheral neutrophil levels are more strongly associated with survival in female with ALS than in male individuals (7). Given that the incidence rate of ALS varies based on both sex and age (17), it is possible that sex- and age-specific immune differences may contribute to these demographic differences. Therefore, the goal of the present study was to examine the role of NK cells in ALS and determine whether sex and age alter the impact of NK cells.

First, NK cells were depleted in SOD1^G93A^ ALS mice, and the effect of sex on survival and neuroinflammation was assessed to determine whether sex-specific immune mechanisms affect the course of disease. Next, in human subjects, NK cell surface marker expression was used to assess NK cell activation and identify changes in NK cell subpopulations (18). NK cell surface marker expression in the peripheral blood of subjects with ALS was examined and compared with those in controls. Markers involved with cytotoxic function (CD38, KIR2DL1, KIR2DL2, NKG2D, NKp30, NKp46), differentiation state (CD45RA, CD57, CD62L), trafficking (CD11a, CD11b, CX3CR1, CXCR3), or antiinflammatory activity (CD27, CD94) were examined, and these data were then stratified by sex and age. Changes in these NK cell metrics were then correlated with changes in the revised ALS functional rating scale (ALSFRS-R), a measure of ALS disease severity, to determine whether longitudinal changes in immune markers associate with ALS progression (4). Finally, these correlations were then stratified by sex and age to determine whether these factors modify the impact of NK cells in ALS. The collective findings suggest that NK cells contribute to ALS progression and that the mechanism underlying their involvement is both sex and age specific.

## Results

### NK cell depletion in ALS mice.

**Figure 1:**
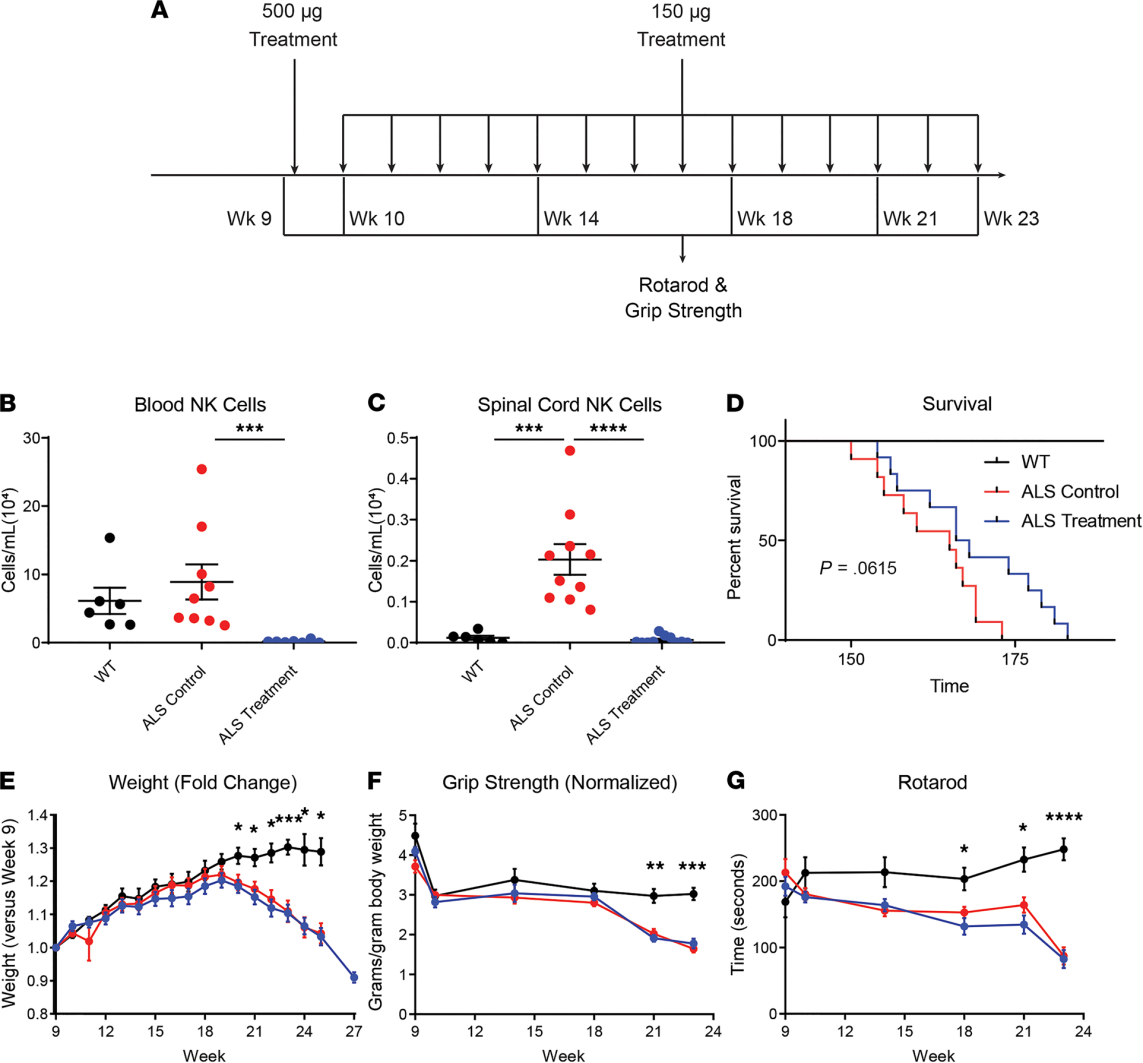
Effects of NK1.1^+^ NK cell depletion on ALS mice. (**A**) SOD1^G93A^ ALS mice were treated with an initial intraperitoneal dose of 500 μg NK cell depleting antibody and subsequent weekly boosters of 150 μg (ALS treatment, blue); a control group of ALS mice was treated with a nonspecific IgG sham treatment (ALS control, red). Both were compared with WT mice (black) mice. Time on rotarod and forelimb grip strength was examined at 9, 10, 14, 18, 21, and 23 weeks of age. (**B**) Levels of NK1.1^+^ NK cells were examined in the peripheral blood of WT (*n* = 6), ALS control (*n* = 9), and ALS treatment (*n* = 7) mice, and (**C**) in the spinal cord (WT, *n* = 6; ALS control, *n* = 10; ALS treatment, *n* = 10) using flow cytometry at the terminal disease endpoint. Blood levels were compared using Kruskal-Wallis due to non-normal distribution, and spinal cord levels using ANOVA. (**D**) Mouse survival was analyzed using Kaplan-Meier to compare WT, ALS control, and ALS treatment mice. (**E**) Fold change in weight, (**F**) grip strength (normalized to body weight), and (**G**) time on a rotarod were analyzed at regular intervals for WT (black; *n* = 6), ALS control (red; *n* = 11), and ALS treatment (blue; *n* = 12) mice. Data at each time point were analyzed using 2-way ANOVA with multiple comparisons. Mean and SEM are displayed. *P < 0.05, **P < 0.01, ***P < 0.001, ****P < 0.0001.

**Figure 2:**
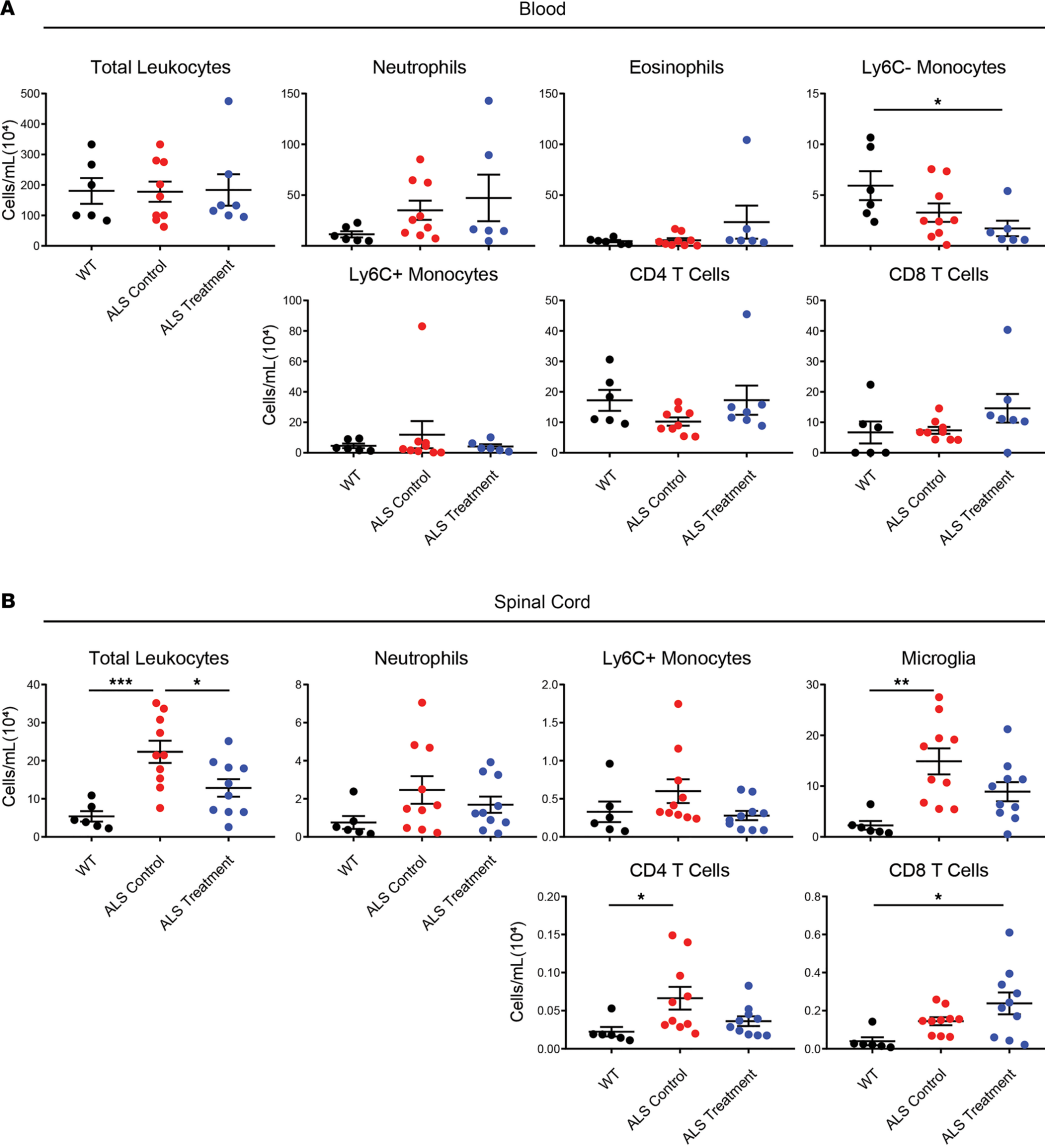
Effects of NK1.1^+^ NK cell depletion on immune cell levels in the blood and spinal cord of SOD1^G93A^ mice. Total immune cells and specific cell populations were examined in (**A**) the peripheral blood of WT (black, *n* = 6), ALS control (red, *n* = 9), and ALS treatment (blue, *n* = 7) mice, and (**B**) the spinal cord (WT, *n* = 6; ALS control, *n* = 10; ALS treatment, *n* = 10). Mean and SEM are displayed. Groups were compared using ANOVA. *P < 0.05, **P < 0.01, ***P < 0.001.

To first examine the role of NK cells in ALS and the impact of sex, NK1.1^+^ NK cells were depleted in SOD1^G93A^ ALS mice using a commercially available depleting antibody (Ultra-LEAF purified anti-mouse NK-1.1 antibody). Mice were injected with an initial anti-NK1.1 antibody dose followed by weekly boosters (Figure 1A; ALS treatment group) and compared with SOD1^G93A^ mice receiving an identical sham regimen of nonspecific IgG antibody (ALS control group) and untreated healthy WT mice. As measured by flow cytometry, the depleting antibody reduced NK1.1^+^ NK cells in the peripheral blood (Figure 1B) and NK cell accumulation in the spinal cord (Figure 1C) compared with sham-treated ALS control mice at the disease terminal endpoint. However, as the NK1.1 marker is expressed on the surface of half of SOD1^G93A^ NK cells (Supplemental Figure 1A; supplemental material available online with this article; https://doi.org/10.1172/jci.insight.147129DS1), only half of all NK cells were depleted by the treatment because NK1.1^–^ NK cells were unaffected (Supplemental Figure 1B). This depletion had a modest but nonsignificant increase in survival (*P* = 0.0615; Figure 1D), and treatment did not prevent loss of weight (Figure 1E), grip strength (Figure 1F), or rotarod agility (Figure 1G). Because peripheral and CNS immune changes were previously observed in ALS mice (19), the impact of NK cell depletion was examined in these locations. Depletion did not affect peripheral blood total leukocyte, neutrophil, eosinophil, Ly6C^+^ monocyte, CD4 T cell, or CD8 T cell levels (Figure 2A); only Ly6C^–^ monocyte levels were affected. In contrast, anti-NK1.1 antibody treatment lowered total leukocyte levels in the spinal cord of ALS treatment mice versus ALS control mice (Figure 2B), and there was a trend toward reduced microglia, Ly6C^+^ monocytes, and CD4 T cells.

**Figure 3:**
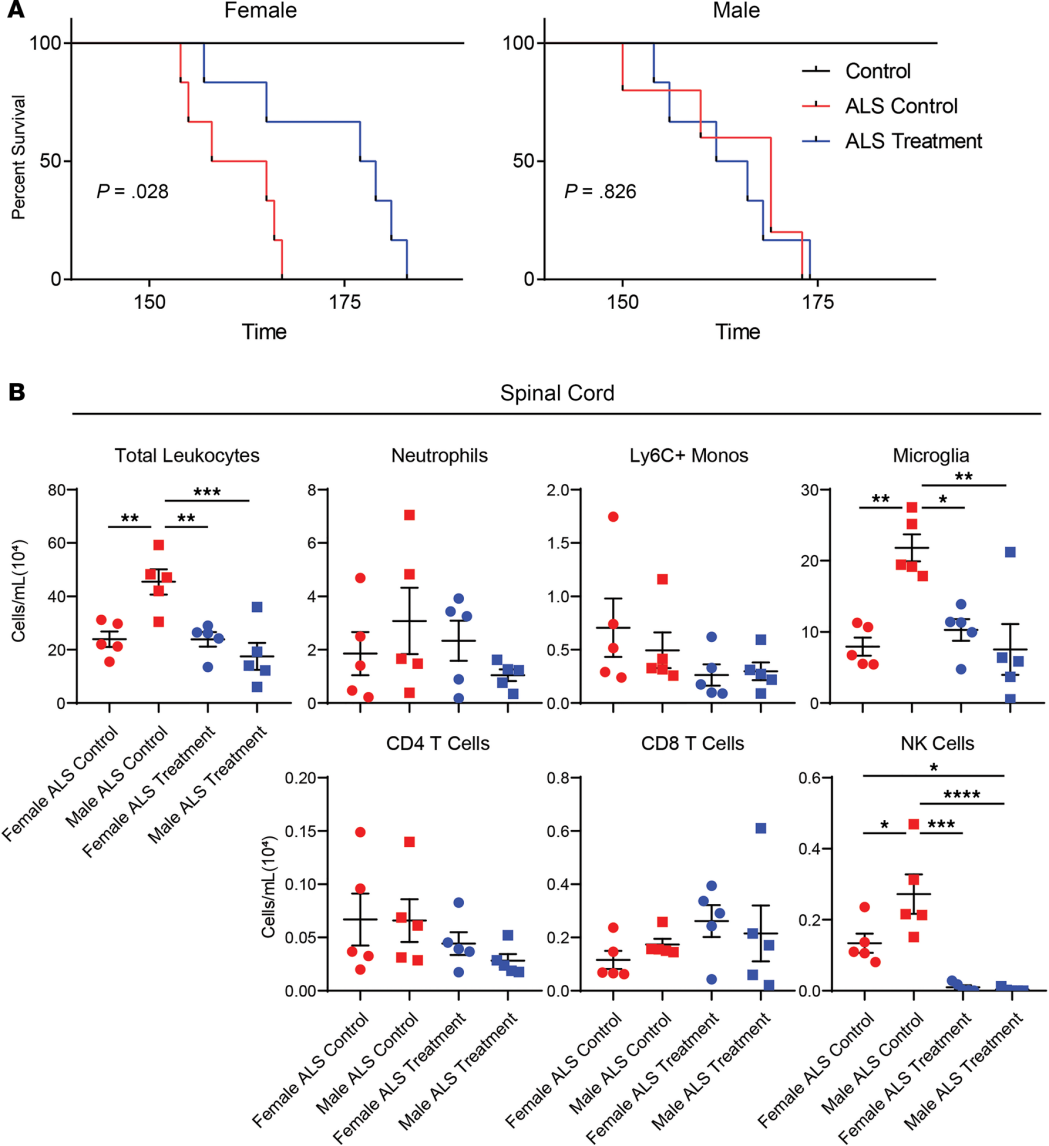
Effects of NK1.1^+^ NK cell depletion in male and female SOD1^G93A^ mice. (**A**) Mouse survival was stratified by sex and analyzed by Kaplan-Meier to compare WT (black; female *n* = 3, male *n* = 3), ALS control (red; female *n* = 5, male *n* = 5), and ALS treatment (blue; female *n* = 6, male *n* = 6) mice. (**B**) Leukocyte accumulation in the spinal cord of ALS control (red; female *n* = 5, male *n* = 5) and ALS treatment (blue; female *n* = 5, male *n* = 5) mice was stratified by sex. Mean and SEM are displayed. Group differences were assessed by ANOVA. *P < 0.05, **P < 0.01, ***P < 0.001, ****P < 0.0001.

Next, the mouse data were stratified by sex, and survival rates and inflammatory changes were reanalyzed to determine whether the impact of NK cells in ALS is sex specific. Strikingly, NK cell depletion increased survival of female ALS mice but produced no survival benefit in male mice (Figure 3A). In addition, whereas NK cell depletion reduced CNS NK cell levels in both male and female mice, the number of CNS microglia were reduced only in male mice. Interestingly, male ALS control mice had more microglia and NK cells in the CNS than ALS control mice, indicating that there are sex-specific immune differences in the spinal cord during natural disease progression (Figure 3B). CNS neutrophil, Ly6C^+^ monocyte, CD4 T cell, and CD8 T cell levels were not affected by NK cell depletion. These findings demonstrate that the CNS immune environment is different in male and female mice during ALS and that NK cell depletion results in a reduction in microglial levels in the CNS in male mice. Despite this reduction in male mice, NK cell depletion only extended the survival of female mice, suggesting that separate immune mechanisms may exist for males and females with ALS.

### NK cell subpopulations and surface marker expression in subjects with ALS.

Based on these results, as well as previous findings showing a sex-specific association between neutrophils and survival (7), NK cell numbers, NK cell subpopulation numbers, and NK cell surface marker expression (Supplemental Table 1; ref. 18) were prospectively analyzed in the peripheral blood and examined for differences based on sex and age. NK cell metrics were assessed in 205 subjects with ALS and 94 control subjects (Table 1); though demographics were similar, subjects with ALS were slightly older, had a higher percentage of males, and consisted of fewer non-Whites compared with controls. A total of 15 detectable NK cell surface markers were examined to quantify NK cell subpopulations with specific proinflammatory and regulatory function or to quantify trafficking, cytotoxicity, and differentiation markers universally expressed on all NK cells (18). Of the 15 markers, 7 were expressed on a fraction of NK cells and used to analyze their subpopulations, whereas 8 were expressed universally on NK cells (Supplemental Table 1). There were no differences in total peripheral NK cell levels in subjects with ALS versus control subjects or in CD57^+^, CD62L^+^, CD94^+^, KIR2DL1^+^, and KIR2DL2^+^ subpopulations (Table 2 and Supplemental Figure 2). However, CD27^+^ (antiinflammation) and CXCR3^+^ (tumor trafficking) NK cells were decreased in ALS. In contrast, there were large increases in expression of NK cell surface markers involved in cytotoxicity and trafficking including CD11a, CD11b, CD38, CX3CR1, NKG2D, and NKp46 in subjects with ALS versus control subjects. Increases in these markers suggest that during ALS, peripheral NK cells are more capable of trafficking to inflammatory sites and dispatching damaged cells.

Based on the sex-specific responses to NK cell depletion in mice, as well as the disparate incidence rates of ALS among demographic groups (17), changes in peripheral NK cell numbers or surface marker expression were compared in men and women or in older and younger subjects. To do so, total NK cell numbers, NK subpopulation numbers, and NK cell surface marker expression values were stratified by sex (men or women) or age (<55 years or >55 years). Fifty-five years of age was selected to ensure that women were beyond the average age range of menopause (51–55 years) in the United States (20). When the cross-sectional data set was stratified, no sex- or age-specific differences in total NK cell numbers, NK cell subpopulations, or NK surface marker expression in the peripheral blood of subjects with ALS were found (data not shown). Together, these results demonstrate that although NK cells show increased activation in the periphery during ALS based on markers of trafficking and cytotoxicity, these markers did not differ based on sex or age when examined using a cross-sectional analysis.

### Correlation between changes in NK cells and changes in ALSFRS-R.

This initial cross-sectional analysis was then expanded to examine the association between changes in NK cell metrics and disease progression as well as the impact of sex and age on these associations. To do so, NK cell metrics were examined at 6-month intervals in a cohort of 92 subjects with ALS (Table 3); this cohort of subjects was taken from the larger cohort of 205 subjects and consisted of subjects who had provided samples at 2 or more time points. For each subject with ALS, changes in NK cells, NK cell subpopulations, and NK cell surface markers were assessed between clinical visits, and a linear mixed-effects (LME) model was used to generate a correlation coefficient that measured the association between change in each NK cell metric and change in ALSFRS-R. A positive correlation coefficient indicates that changes in NK cell marker levels and ALSFRS-R occur in the same direction, and a negative correlation coefficient indicates they occur in the opposite direction.

Changes in total NK cells and most subpopulations did not correlate with change in ALSFRS-R. However, there was a modest negative correlation between changes in CXCR3-expressing NK cells to ALSFRS-R changes (Table 4). In contrast, changes in NK cell trafficking and cytotoxicity marker expression levels were highly correlated with ALSFRS-R changes. Changes in CD11a, CD11b, CD38, CX3CR1, NKG2D, and NKp46 — all of which were highly upregulated in subjects with ALS compared with controls (Table 2) — positively correlated with changes in ALSFRS-R. In addition, changes in NKp30 — which was not altered in the initial cross-sectional case versus control analysis — strongly and negatively correlated with changes in ALSFRS-R.

**Figure 4:**
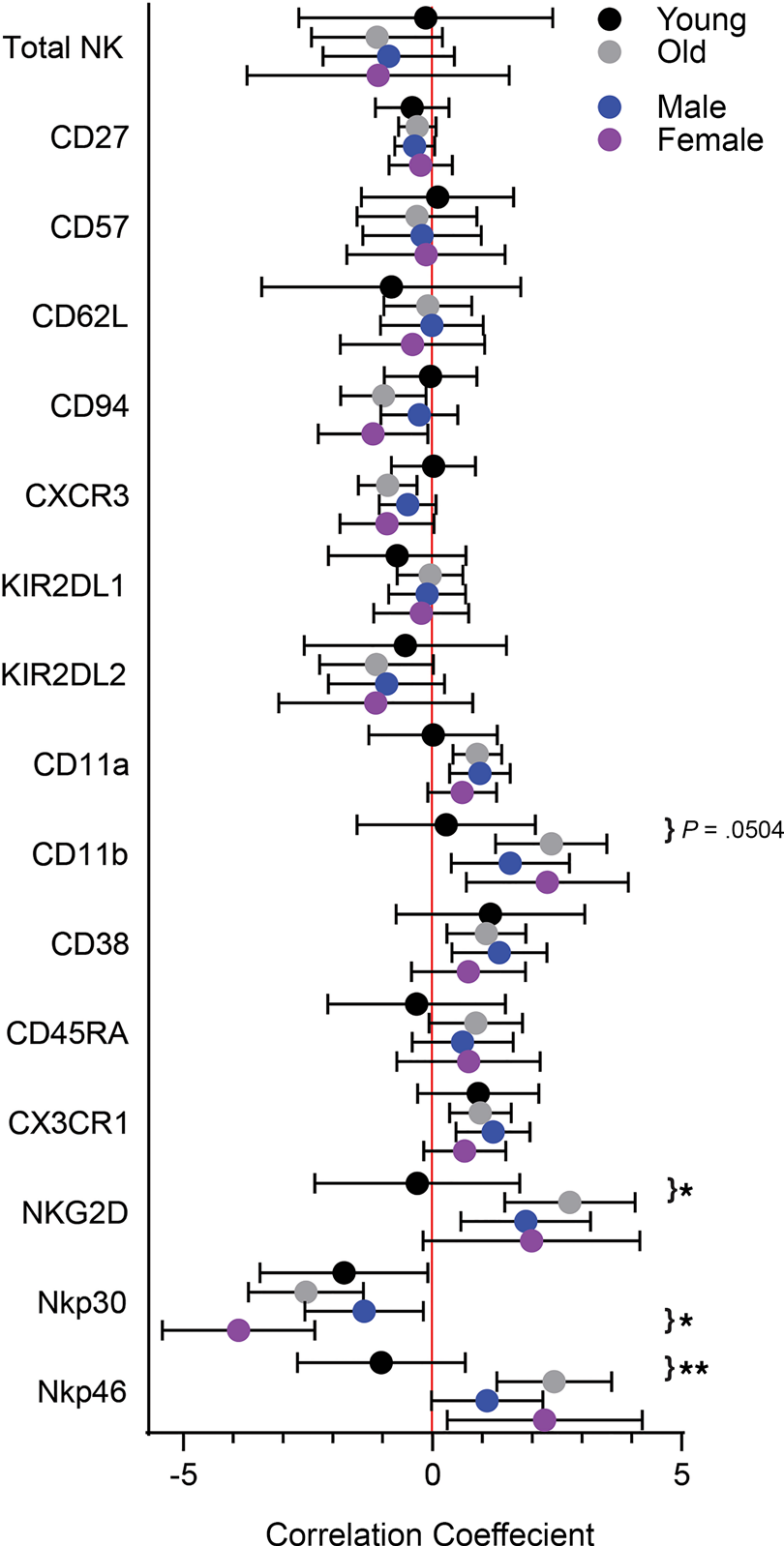
Correlation between changes in NK cell metrics and changes in ALSFRS-R stratified by age or sex. Correlation coefficients were obtained from the (LME) model (*n* = 92). Subjects were classified into young (≤55 years of age, black circles, *n* = 26) vs. old (>55 years of age, gray circles, *n* = 66), or male (blue circles, *n* = 58) vs. female (purple circles, *n* = 34). Circles indicate the mean and whiskers indicate 95% confidence intervals. Differences between young and old or between male and female were assessed by testing the NK cell metric by sex interaction or NK cell metric by age interaction, respectively, in the LME model, *P < 0.05, **P < 0.01. LME, linear mixed-effects; ALSFRS-R, Revised ALS Functional Rating Scale.

As in the cross-sectional analysis, correlations were then stratified by sex (men or women) or age (young, <55 years; old, >55 years). The positive correlation between NKG2D and ALSFRS-R changes was higher in older versus younger subjects as was the association between NKp46 and ALSFRS-R changes (Figure 4); a similar trend was observed with CD11b, though correlation differences only approached significance (*P* = 0.0504). In parallel, there were differences in correlation coefficients between men and women. In particular, the negative association between NKp30 and ALSFRS-R changes was more pronounced in ALS women versus men. Together, these data indicate there are strong correlations between NK cell surface marker changes and ALSFRS-R changes (and by extension disease progression) which differ by sex and age in subjects with ALS.

## Discussion

The present study examined whether NK cells contribute to ALS progression and whether this contribution is sex and age specific. In ALS mice, depleting NK cells extended survival in female but not male mice. In addition, male ALS control mice had higher levels of microglia in the CNS than female ALS control mice; this was ameliorated by NK cell depletion. In human subjects, NK cells showed increased expression of surface markers involved in trafficking and cytotoxicity but not in differentiation. Further, changes in trafficking and cytotoxicity markers associated with changes in ALSFRS-R in an age- and sex-specific manner. These findings implicate NK cells in ALS progression, but they also demonstrate that sex- and age-specific immune mechanisms likely contribute to ALS pathogenesis.

Previous studies have linked immune changes to ALS progression both in humans and mouse models (2, 4, 11, 19, 21). Depletion of specific immune populations can slow or accelerate disease in mouse models (5, 12, 22), and multiple immune cell types are dysregulated in mice and humans with ALS including CD4 T cells (22–24), regulatory T cells (25, 26), and monocytes (5, 24, 27). NK cells are immune cells of particular interest because motor neurons are vulnerable to NK cell cytotoxicity during ALS (10). In addition, there are altered NK cell levels in the periphery of humans with ALS (4, 11), and a recent study found that NK cells accumulate in the CNS of subjects with ALS, colocalize with motor neurons, and contribute directly to microglial inflammation in the CNS via the expression of IFN-γ (13). These observations make a strong case that NK cells play a central role in ALS progression.

This case is bolstered by the current finding that multiple NK cell surface markers are upregulated in subjects with ALS compared with controls: CD11a, CD11b, CD38, CX3CR1, NKG2D, and NKp46 are all highly upregulated during disease. These proteins are incredibly important in NK cell functions such as trafficking (CD11a, CD11b, CX3CR1) and cytotoxicity (CD38, NKG2D, NKp46; ref. 18), allowing NK cells to migrate to infected or damaged cells and eliminate them. CX3CR1 is particularly important for NK cell migration to the CNS (28), and the NKp family of proteins, including NKp30 and NKp46, are crucial for triggering NK cells’ elimination of target cells (29). Interestingly, no differences were observed in expression of CD45RA, a surface marker associated with differentiation. The lack of increased NK cell differentiation, coupled with similar peripheral NK cell levels in subjects with ALS and control subjects, suggests that NK cell capacity for trafficking and cytotoxicity has a greater impact on ALS pathogenesis than total NK cell numbers.

This idea is supported by the longitudinal data that found that changes in surface markers, including NKp30, correlated with changes in ALSFRS-R, whereas changes in total NK cell levels did not. Interestingly, changes in trafficking and cytotoxicity markers positively correlated with decreases in ALSFRS-R, meaning that as the disease progresses, there is a parallel decrease in expression of these markers. The exception was NKp30, a cytotoxicity marker, which increased as disease progressed. These findings suggest that the majority of NK cell activation occurs early in disease when the ALSFRS-R score is higher and likely resolves as motor neurons die. The data also suggest that separate NK mechanisms may contribute to disease progression: unlike other surface markers, NKp30 expression increases as disease progresses, meaning that NKp30 may drive cytotoxicity in later stages of ALS. These observations are consistent with previous findings (4) that the role of the immune system in ALS changes over time: peripheral neutrophil levels are negatively associated with ALSFRS-R changes (increasing as disease worsened), whereas CD4 T cell levels are positively associated with ALSFRS-R (decreasing as disease worsened; ref. 4).

More striking than these associations, however, are the sex- and age-based differences in correlation coefficients: correlation between cytotoxicity markers NKG2D or NKp46 and ALSFRS-R were higher in older subjects, and correlation between cytotoxicity marker NKp30 and ALSFRS-R was higher in women. These data suggest specific NK cell activation pathways, such as those triggered via NKG2D and NKp46 signaling, may play a greater role in driving disease progression in older individuals, and that NKp30 signaling may play a greater role in women. Indeed, sex-based differences in immune responses have been reported in other diseases ranging from influenza (30) to asthma (31) to malaria (32). These differences can also impact neurodegenerative diseases, such as Alzheimer’s disease (16), and in ALS, peripheral neutrophil levels have a greater association with survival in women than men (7). Similarly, a recent prospective clinical study found that increased overall immune levels in the periphery were associated with shorter survival in women with ALS than men, though these differences did not reach statistical significance (33).

These observations are further supported by the mouse data, in which untreated male mice had increased microglial and NK cell inflammation in the CNS compared with female mice. Despite this, NK cell depletion extended the life span of female but not male mice, further demonstrating sex-specific immune differences in ALS. These results are consistent with a previous study that found that the deletion of CX3CR1 in the microglia of ALS mice accelerated disease, but only in male mice (34). Combined with the human data, these findings in mice suggest that sex differences contribute to alternative immune mechanisms in males and females with ALS (17, 35).

However, although the data indicate sex- and age-based immune factors affect ALS progression, the underlying mechanisms driving these differences are not known. The most likely source of the observed age and sex differences is the influence of sex hormones because plasma levels of testosterone and estrogens differ between men and women and change with age or onset of menopause (36, 37). Though understudied, multiple reports also suggest that hormones can directly alter immune cell function including the function of NK cells (38). In the current study, however, the overall numbers of NK cell subpopulations and expression of surface markers were not different in men or women with ALS, nor were they different in younger or older subjects. These data therefore suggest that the sex- and age-specific effects of the NK cell surface markers, i.e., NKp30, NKp46, and NKG2D, did not likely occur in the periphery but in the CNS.

Previous studies support this contention and have shown that the CNS immune microenvironment is distinct in men and women (39, 40) as well as in the old and the young (41). Though the underlying mechanisms driving these differences are not completely understood, the expression level of prostaglandin E2 (PGE2) in the CNS might account for the sex-specific differences in both mice and humans with ALS. PGE2 has multiple functions in the body including sexual development; in the CNS, its expression is driven by testosterone signaling and plays a central role in the development of male sexual behavior (40). It may also directly contribute to neuronal damage in ALS: not only are PGE2 levels increased in the cerebrospinal fluid of subjects with ALS (42), but also the disruption of the PGE2 signaling pathway slows disease progression and extends the life span of ALS mice (43). Yet, PGE2 is also a potent regulator of the immune system that suppresses destructive immune responses (44). Thus, during ALS there may be 2 separate but competing CNS mechanisms that contribute to neurodegeneration. In males, increased PGE2 levels would contribute directly to neurodegeneration while suppressing immune cytotoxicity. As seen in the male ALS mice, this would result in increased cellular accumulation because cells continually traffic to a site of chronic, unresolved inflammation (45). In contrast, females would be protected from direct PGE2 cytotoxicity, but the lack of PGE2-mediated regulation would result in a more proinflammatory immune environment. Immune cells trafficking to the CNS, including NK cells, would therefore respond more aggressively to damaged tissue in females. This might explain why NKp30 expression levels are associated more strongly with ALS progression in women and why neutrophils have a greater impact on survival in women during disease (7). However, further studies will need to be conducted to determine these mechanisms with certainty.

Alternatively, other immune cell populations such as neutrophils may contribute to differing NK cell function as well. For instance, previous reports have found that NK cells can regulate neutrophil function (46) and vice versa (47). Given that neutrophils also have a sex-specific effect on ALS survival (7), it is therefore possible that interactions between NK cells and neutrophils may influence ALS progression. For example, numerous neutrophil-derived factors, such as cathepsin G and elastase, can drive NK cell cytotoxicity (48), and at least one study has found that increasing levels of female sex hormones induce neutrophil expression of these factors (49). Unfortunately, although studies have found that sex and age can both impact the function of the immune system (50, 51), to date very little research has examined whether these factors impact the interaction between different types of immune cells. Additional studies will be needed to determine whether interactions between immune cell populations contribute to ALS progression rates and whether this contribution is affected by sex or age.

Regardless of the underlying mechanisms, the findings of the current study are critical to the design and interpretation of future ALS clinical trials, particularly those using immunotherapy. Though early clinical trials using immunosuppression failed (52), there has been a resurgent interest in immunomodulatory treatment for ALS because the complex interplay between peripheral and CNS immunity is better understood. Multiple immune-based ALS clinical trials are currently ongoing, and there is the strong likelihood of additional immunotherapies being tested in the future. Despite this renewed interest, however, few studies have examined the impact of sex or age on *any* aspect of ALS pathogenesis, much less the underlying immune mechanisms. Given the disparate ALS development (17) and progression rates (1) between men and women and between the young and the old, sex and age should be accounted for when designing future ALS clinical trials. Finally, although the impact of sex and age have not been thoroughly studied in ALS, this omission is not unique to ALS because the roles of sex and age have been overlooked in many neurological diseases. Rates of neurodegenerative disorders such as Alzheimer’s disease differ by sex (53) and age (54), and sex-based differences are even more pronounced in immune-based neurological diseases such as multiple sclerosis (55). Despite this, few clinical trials, particularly immunotherapy-based trials, account for sex or age (56–58). The current study therefore represents an important step forward in understanding how sex and age contribute to ALS and suggests similar research is warranted in multiple other neurodegenerative diseases.

Despite the importance of the findings, this study does have limitations. First, the mouse work is limited by relatively small sample sizes; however, the findings are nonetheless supported by the observations in human subjects. In human subjects, all possible NK surface markers and subpopulations were not measured, only markers likely to be most crucial to function; additional markers would not likely lead to different associations, but this was not tested. Moreover, although subjects with ALS were sampled at multiple time points, control subjects were only sampled once, and there were also demographic differences between subjects with ALS and control subjects that could potentially impact observed surface marker differences. However, we observed no differences in NK cell markers between subjects with ALS when these data were stratified by sex or age, suggesting that demographic differences did not impact the analysis. Next, although subjects with ALS were studied relatively early in disease, it is unclear if the findings would be different if these measures were obtained prior to diagnosis, though this is a limitation for all ALS studies. Finally, there were relatively low numbers of young subjects with ALS in the correlative analysis, and there were age differences between younger men with ALS (mean = 45, *n* = 13) and younger women with ALS (mean = 51; *n* = 7). Therefore, when stratifying the data by sex, the results may be impacted by older subjects, and when stratifying by age, the results may be impacted by men. Ideally, the correlation analysis would be stratified by both age and sex simultaneously; however, the relatively low number of young subjects with ALS, particularly women, precludes this analysis, and future studies will be required.

In conclusion, the data show that NK cells likely participated in ALS progression but that their involvement was sex and age specific. These findings are consistent with previous studies that have noted sex-specific immune differences in neurodegenerative diseases, including ALS (7, 16). A more comprehensive analysis of the impact of sex and age on ALS, as well as an exploration of the underlying mechanisms driving sex- and age-specific immune mechanisms, is therefore critically needed. Moreover, sex and age should be accounted for when examining the immune system and related therapeutic targets in ALS.

## Methods

### Animals, sample collection, and survival analysis.

Thirty-day-old male and female SOD1^G93A^ ALS mice (B6.Cg-Tg(SOD1*G93A)1Gur/J; stock 004435) and WT control littermates (The Jackson Laboratory) were housed in a dedicated facility and fed 5L0D chow (LabDiet). Each animal was genotyped by PCR with tail genomic DNA using a protocol from The Jackson Laboratory. For initial assessment of NK cell depletion efficacy, 60-day-old C57BL/6 mice (stock 000664) were purchased from The Jackson Laboratory.

### NK cell depletion.

SOD1^G93A^ mice were separated into 2 groups; 1 group received intraperitoneal injections with a commercially available NK1.1-depleting antibody (Ultra-LEAF purified anti-mouse NK-1.1 antibody, clone PK136, catalog 108762; BioLegend) and 1 was treated with a nonspecific, nondepleting sham IgG control antibody (Ultra-LEAF purified mouse IgG2a κ isotype control, catalog 400290; BioLegend). Treatment began at 60 days of age with an initial dose of 500 μg followed by weekly 150 μg boosters. Depletion was verified in an initial cohort of 6 C57BL/6 mice — which express NK1.1 on all NK cells — after 4 or 8 weeks of treatment using flow cytometry with antibodies against both NK1.1 and CD49b (Supplemental Figure 3).

### Motor behavior phenotyping.

Mice were weighed weekly and assessed for grip strength and agility on a rotarod over time as previously described (19). For forelimb grip strength, a grip strength meter with a single sensor and a standard pull bar and software were used (Columbus Instruments). Motor function and agility were assessed using a Rotarod series 8 instrument (IITC Life Science).

### Collection of immune cells from mouse tissue.

ALS mice were euthanized by lethal pentobarbital injection (Vortech Pharmaceutical) at the terminal disease endpoint defined by when the animals were no longer able to right themselves within 10 seconds. After euthanasia, blood and spinal cords were collected for immunophenotyping as previously described (19). For half of the ALS mice, an age-matched WT control animal was also sacrificed. Because CNS inflammation was most pronounced in later stages of disease in SOD1^G93A^ mice (19), immune changes were assessed at the terminal endpoint of disease to more easily assess the impact of NK cell depletion.

### Human study population and sample collection.

Subjects with a diagnosis of ALS or ALS with frontotemporal dementia meeting suspected, possible, probable laboratory supported, probable, or definite ALS by El Escorial criteria were recruited during clinical visits at the University of Michigan Pranger ALS Clinic. Healthy controls without a neurodegenerative disorder, chronic inflammatory disease, collagen vascular disease, or immunomodulatory medication use were recruited through the University of Michigan Institute for Clinical & Health Research. Samples were collected between June 2016 and October 2019, and all subjects were without fever or infectious illness at the time of blood collection. Samples from subjects with ALS were roughly every 6 months; control samples were collected once. For subjects with ALS, ALSFRS-R score was determined at each clinical visit. Blood samples were obtained by peripheral venipuncture and processed within 2 hours of collection as previously described (4).

### Flow cytometry.

Immune cells from human subjects and mice were processed identically prior to staining; cells were plated at a density of ≤10^6^ cells/25 μL in flow cytometry buffer (FCB) in 96-well round-bottom plates (Corning), and Fc receptors were blocked with 10 μg/mL human or mouse TruStain FcX blocking solution (BioLegend). Cells were stained with antibody cocktails in 50 μL in the dark at 4°C for 30 minutes, washed twice with FCB, resuspended in 185 μL of BD Stabilizing Fixative (BD Biosciences), and transferred to polystyrene tubes (12 × 75 mM; BD Biosciences) for analysis. A total of 2 × 10^4^ to 2 × 10^5^ events were acquired on a BD LSRFortessa flow cytometer with FACSDiva software (BD Biosciences) and analyzed by FlowJo software.

### Mice.

Fluorophore-conjugated antibodies used as follows: BV421-CD8 (catalog 100738), FITC-CD3 (catalog 100204), FITC-Ly6C (catalog 128006), PE-NK1.1 (catalog 108708), PerCP-5.5-CD3 (catalog 100326), PerCP-5.5-CD19 (catalog 115532), APC-CD45 (catalog 103112), PE/Cy7-CD49b (catalog 108921), PE/Cy7-Ly6G (catalog 127617), APC/Cy7-CD4 (catalog 100414), and APC/Cy7-CD11b (catalog 101226) (BioLegend). Separate gating strategies identified immune cells in the peripheral blood (Supplemental Figure 4) and spinal cord (Supplemental Figure 5) as previously described (19). In the spinal cord, monocyte and microglia populations were distinguished based on CD45 levels because microglia have been shown to have low CD45 expression (59).

### Humans.

Human NK cells were identified within the total immune population as CD3^–^ (catalog 300306), CD56^+^ (catalog 318318), CD16^+^ (catalog 302028), and CD14^–^ (catalog 367108) (BioLegend) (Supplemental Figure 6). All other immune populations were eliminated from the analysis. Markers used to identify NK cell subgroups or surface marker expression were all stained with BV421-, PE-, or APC-tagged antibodies (BioLegend) to prevent overlap between emission spectra (Supplemental Table 1). For each surface marker a fold–median fluorescence intensity (fold-MFI) value was generated by dividing the MFI of the surface marker by the MFI of a color-matched, nonspecific IgG-negative control antibody. This was done to minimize the impact of cytometer voltage change over the course of the multiyear study.

### Human NK subgroups and surface markers.

Differences in NK cell numbers and subpopulation numbers between control and ALS samples were calculated by comparing mean values for each population: percent change in total numbers = (NK number^ALS^ – NK number^Control^)/NK number^Control^. Differences in NK cell surface marker expression between control and ALS samples were calculated by comparing the median fold-MFI for surface markers: percent change in MFI = [(fold-MFI^ALS^ – 1) – (fold-MFI^Control^ – 1)]/(fold-MFI^Control^ – 1). Subjects with ALS provide longitudinal samples at approximate 6-month intervals corresponding to clinical visits. Data from multiple clinical visits were averaged for each subject to ensure each subject was equally represented in the initial cross-sectional analysis and that subjects with multiple clinical visits were not overrepresented (4). Differences between ALS and control subjects were assessed using Student’s 2-tailed *t* test for normally distributed, and Mann-Whitney *U* for nonnormally distributed data. Shapiro-Wilk assessed normality (60). Analyses were performed using Prism.

### NK cell associations with ALSFRS-R.

A LME model assessed the association between log-transformed NK cell surface markers and ALSFRS-R score at peripheral immune cell collection in individual subjects. A random effect was included in the model to account for the correlation between repeatedly measured NK cell markers and ALSFRS-R scores on the same subject. A regression coefficient (i.e., the slope parameter) was estimated from the LME model to assess the association between changes in individual cell markers and changes in ALSFRS-R. To assess the association stratified by age (≤55 or >55 years old) or sex, the interaction of each NK cell marker by the subject’s age or sex, respectively, were tested in the LME model separately. Specific contrasts from individuals were derived from the LME model and stratified by sex or age to determine if the interaction effects were significantly different between groups. Analyses were performed using R software (version 4.0.3).

### Statistics.

Kaplan-Meier curves were used to assess survival differences between groups. NK cell levels in the periphery and CNS were compared between groups using a nonparametric Kruskal-Wallis test because data were not normally distributed as assessed using Shapiro-Wilk (60). For physiological phenotyping (weight, grip strength, rotarod performance), 2-way ANOVA with multiple comparisons was used to compare the 3 groups at each time point, because these data were normally distributed. Two-way ANOVA was used to analyze peripheral and CNS immune cell levels. Analyses were performed using Prism (GraphPad, version 8.0.0). ALS versus control demographics were compared using Wilcoxon rank-sum test for continuous data, and Fisher’s exact or χ^2^ test based on cell counts, for categorical data. Differences between ALS and control subjects were assessed using Student’s 2-tailed *t* test for normally distributed, and Mann-Whitney *U* for non-normally distributed data. Shapiro-Wilk assessed normality (60). Analyses were performed using Prism. *P* values of less than 0.05 were considered significant.

### Study approval.

Mouse studies were performed in accordance with IACUC-approved protocols at the University of Michigan (approval PRO00008431). Mouse studies were conducted in accordance with the United States Public Health Service’s policy on Humane Care and Use of Laboratory Animals. Human subjects provided oral and written informed consent and the study received ethics board approval by the University of Michigan Medical School Institutional Review Board (HUM00028826).

## Author contributions

BJM, FEM, SAG, and ELF designed the overall study. For human studies, BJM, SAG, and ELF wrote the IRB protocol. SAG and ELF recruited subjects with ALS for the study. BJM, JPF, CEP, KDR, SAG, and ELF designed the specifics of the human clinical study. BJM, JPF, CEP, KDR, CHP, and SDI processed peripheral blood, counted cell numbers, and stained cells for analysis. BJM and JPF analyzed NK cells using flow cytometry. BJM performed statistical analysis of surface marker comparisons between control subjects and subjects with ALS. LZ performed the correlative analysis of surface markers and examined demographic-specific differences. For mouse studies, BJM, KDR, and FEM designed the specifics of the mouse trial, prepared NK cell depletion treatments, administered treatments, and performed physical phenotyping. BJM, KDR, and FEM isolated peripheral and CNS immune cells for analysis. BJM ran flow cytometry, analyzed the mouse data, and performed statistical analyses. BJM, JPF, CEP, KDR, FEM, CHP, SDI, LZ, SAG, and ELF wrote the manuscript with substantial input from all authors.

## Supplementary Material

Supplemental data

## Figures and Tables

**Table 1:**
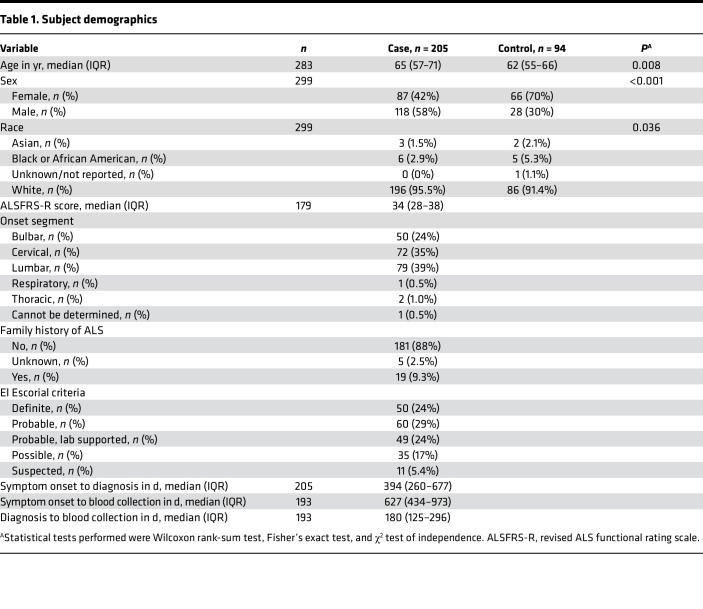
Subject demographics

**Table 2:**
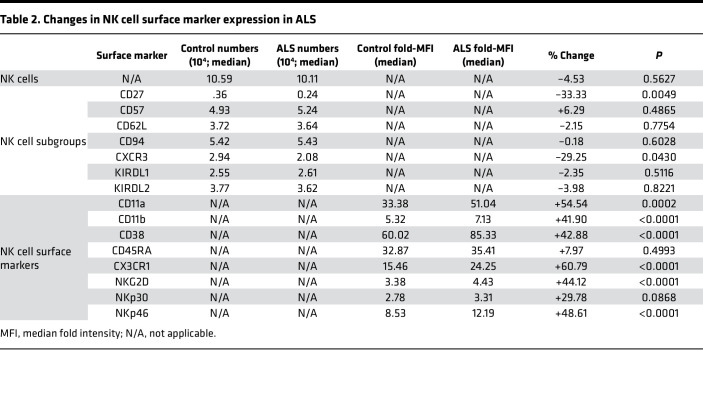
Changes in NK cell surface marker expression in ALS

**Table 3:**
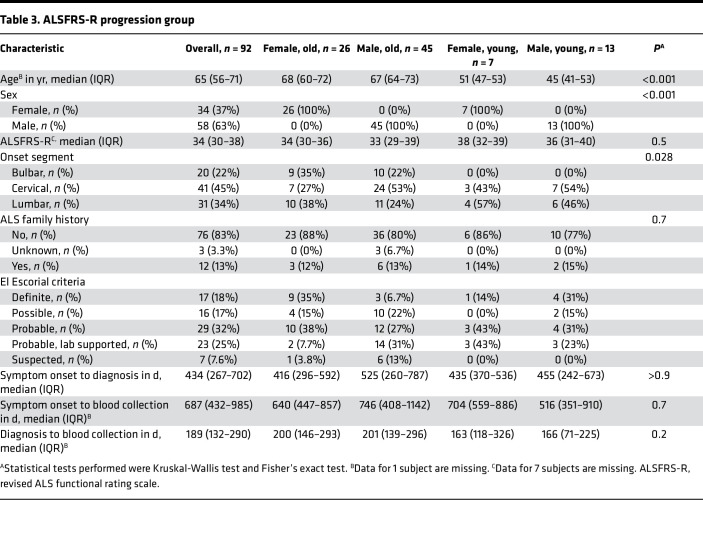
ALSFRS-R progression group

**Table 4:**
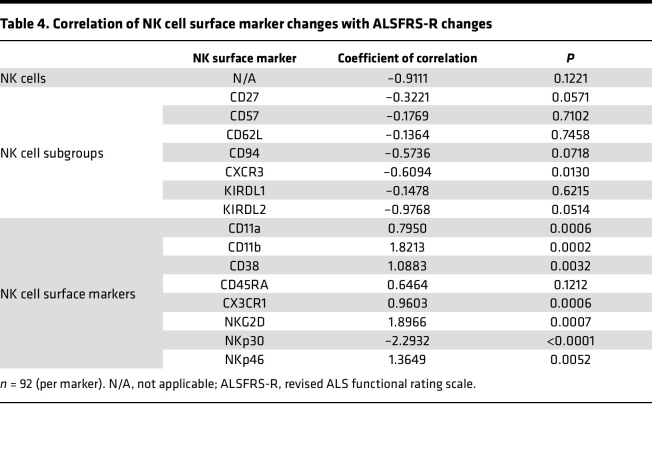
Correlation of NK cell surface marker changes with ALSFRS-R changes

## Version 1 05/11/2021

In-Press Preview

## Version 2 06/08/2021

Electronic publication

## Version 3 06/20/2023

Kristen D. Pawlowski's name was updated
