# Supplementary material for: NK cells associate with ALS in a sex- and age-dependent manner
Source: JCI Insight. 2021 Jun 8;6(11):e147129. doi: 10.1172/jci.insight.147129 (PMC8262328; doi:10.1172/jci.insight.147129)
Supplement: Supplemental data [file jciinsight-6-147129-s009.pdf]

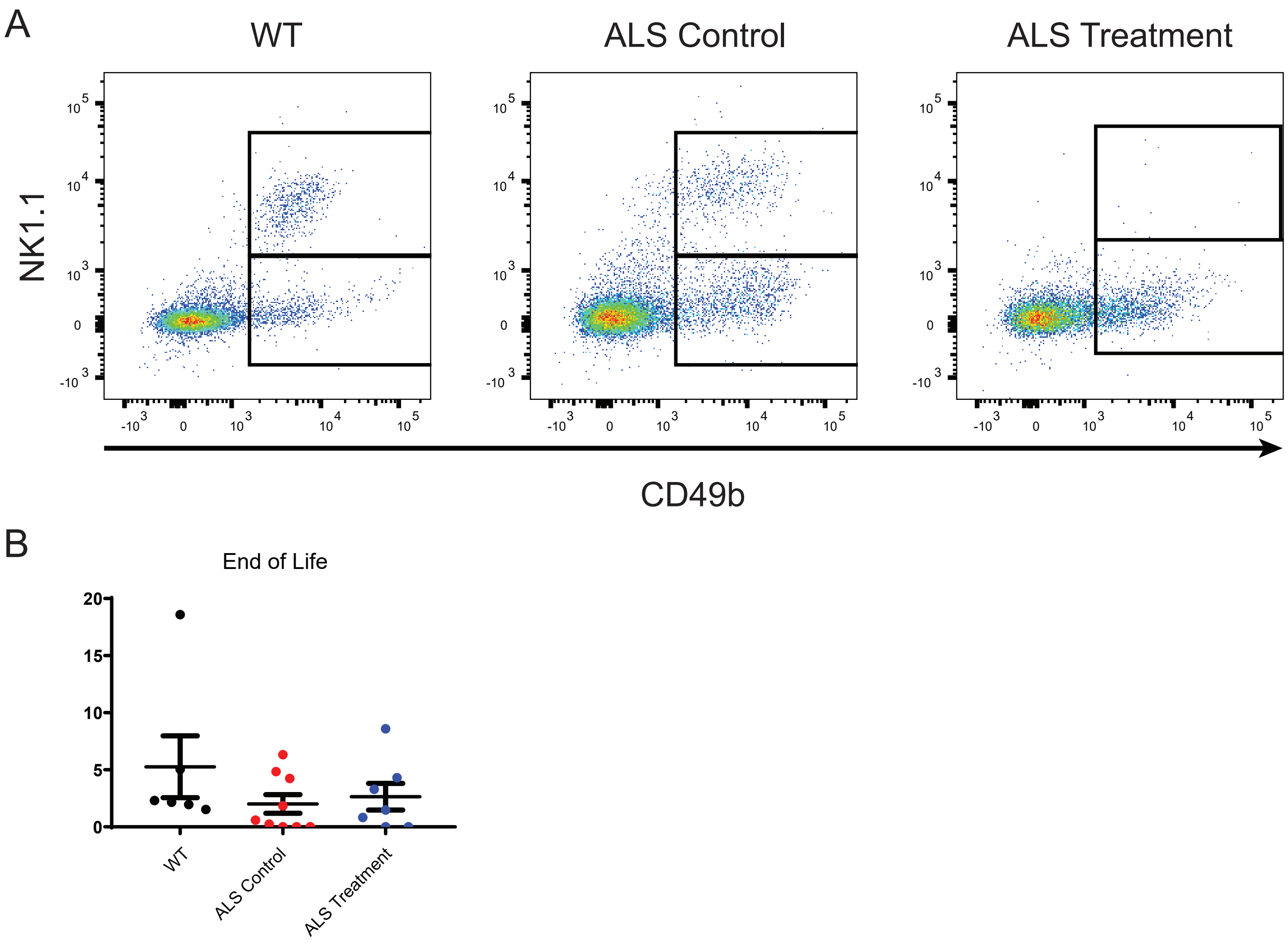


**Supplementary Figure 1.** NK cell depletion and CD49b+ NK cell levels in ALS mice. (A) A representative plot of NK cell NK1.1 and CD49b expression in control mice (Control), ALS mice receiving sham non-specific IgG control antibody (ALS IgG), and ALS mice receiving NK1.1-depleting antibody (Treatment group) is shown. (B) Quantification of peripheral NK1.1- CD49b+ NK cells in Control (black, n = 6), ALS IgG (red, n = 9), or Treatment (blue, n = 7) mice at end of life. Mean and SEM are displayed; ANOVA was used to assess significance.

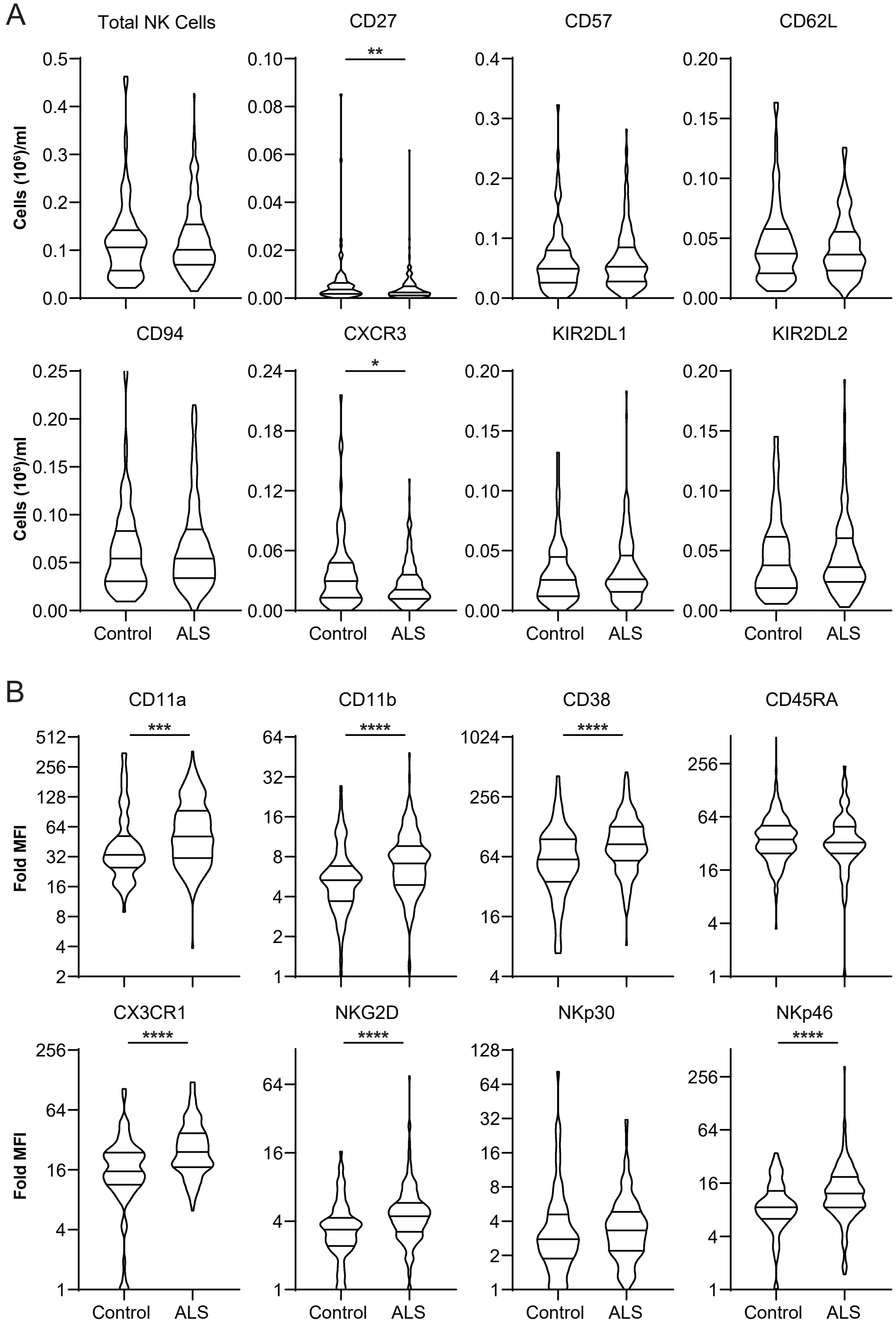


**Supplementary Figure 2.** NK cell populations and surface marker expression in the peripheral blood of control and ALS study subjects. (A) Total NK cell numbers and the numbers of specific NK cell subpopulations was examined in control (n = 94) and ALS (n = 205) subjects using flow cytometry. (B) Expression levels (MFI) of NK cell-associated surface markers was analyzed using flow cytometry in control (n = 94) and ALS (n = 205) subjects. MFI for each marker was normalized to the MFI of a non-specific IgG control to generate a fold-increase in MFI. Data are presented in log2 for ease of interpretation. For ALS subjects who provided samples over multiple visits, data from all visits was averaged to generate a single value per subject. For both NK numbers and surface expression horizontal lines indicate mean and SEM. Control and ALS were compared using Mann-Whitney. *$p < .05$, **$p < .01$, ***$p < .001$, **** $p < .0001$

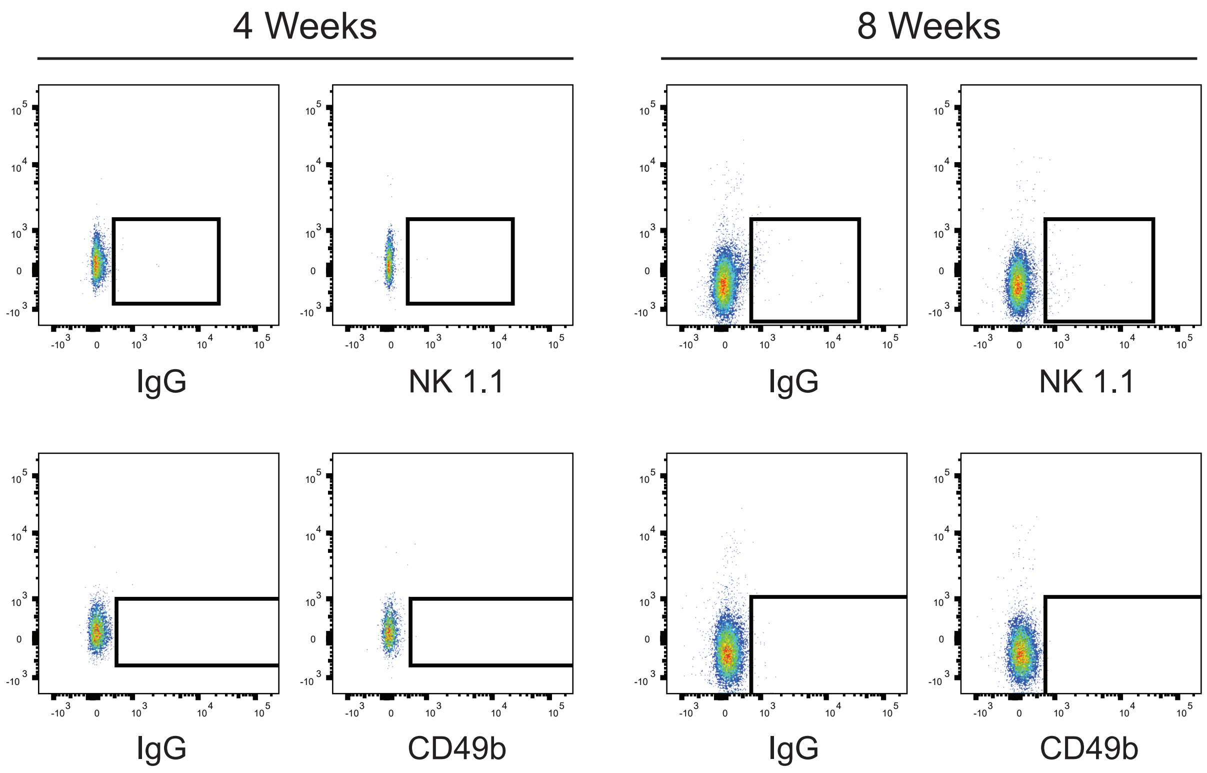


**Supplementary Figure 3.** NK cell depletion efficacy in C57BL/6 mice. C57BL/6 mice were treated for 4 and 8 weeks with NK1.1-specific depleting antibody and peripheral blood leukocyte expression of NK1.1+ and CD49b+ NK cells were assessed using flow cytometry. Non-specific control IgG expression is also shown. For each time point n = 3 mice; panels are representative for each time point.

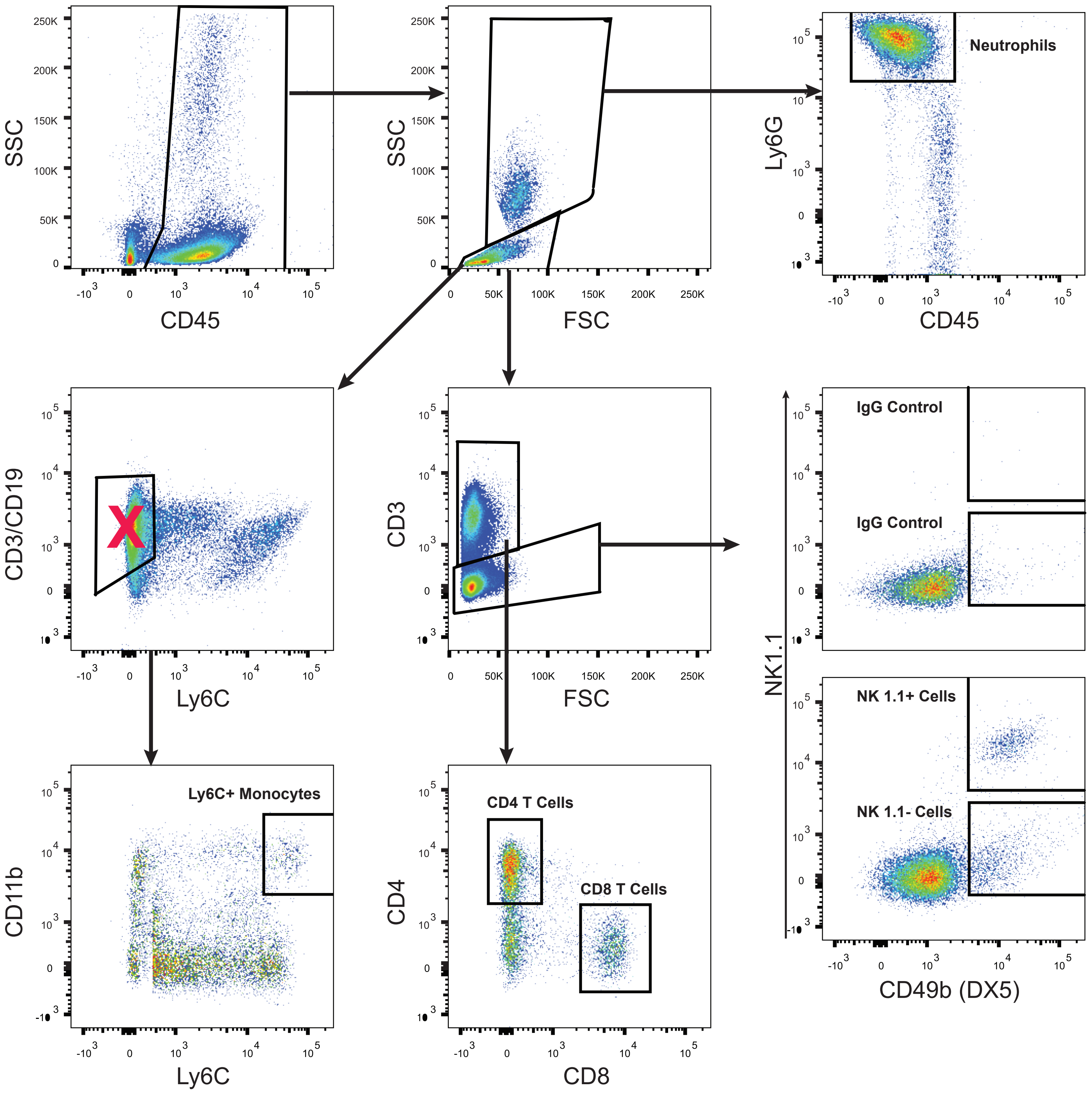


**Supplementary Figure 4.** Gating strategy for leukocytes in peripheral blood in mice. Panels of fluorescently labeled antibodies were used to identify immune cell populations via flow cytometry. Following elimination of doublets, immune cells were identified using a combination of surface marker stains and forward scatter- and side-scatter profiles. CD45 was used to identify leukocyte populations and eliminate residual red blood cells. Neutrophils were then identified via side scatter profile and Ly6G expression. Forward scatter-low cells were used to identify other populations. After gating out CD3+ or CD19+ cells, monocyte populations were identified using CD11b and Ly6C. CD3+ cells were identified as either CD4 T cells or CD8 T cells. CD3- cells were examined for NK1.1 and CD49b expression to identify NK cells. A stain using control IgG was used to calculate and remove background counts.

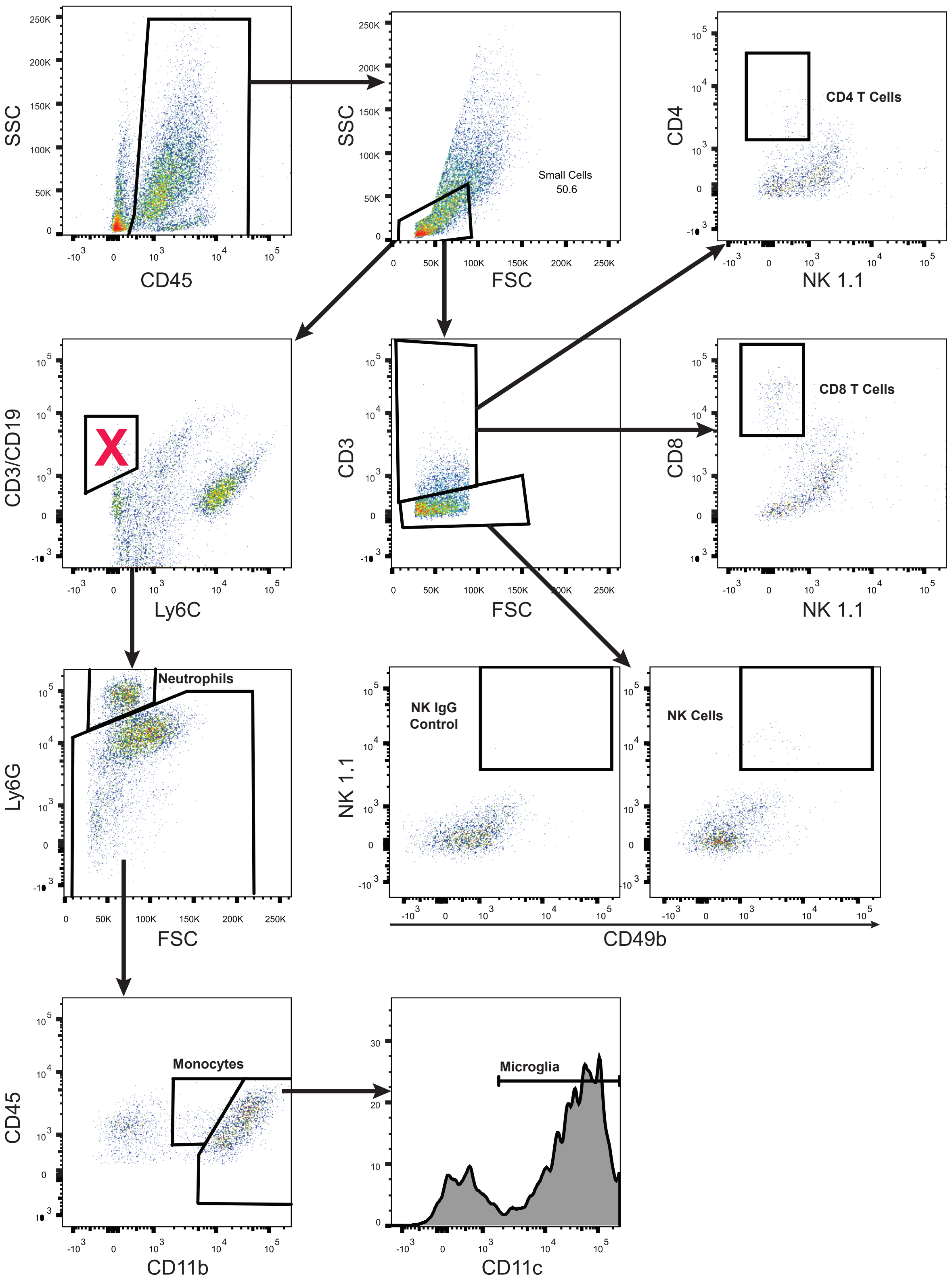


**Supplementary Figure 5.** Gating strategy for leukocytes in spinal cord in mice. Panels of fluorescently labeled antibodies were used to identify immune cell populations via flow cytometry. Following elimination of doublets, immune cells were identified using a combination of surface marker stains and forward scatter- and side-scatter profiles. CD45 was used to identify leukocyte populations and eliminate residual red blood cells, astrocytes, and neuronal debris. Myeloid cells were identified by gating out CD3+ or CD19+ cells, monocyte populations were identified using CD11b and Ly6C. Neutrophils were then identified via side forward profile and Ly6G expression. Forward scatter-low cells were further analyzed for CD11b and CD45 expression to identify monocytes and microglia. Activated microglia were identified using CD11c. CD3+ cells were identified as either CD4 T cells or CD8 T cells. CD3- cells were examined for NK1.1 and CD49b expression to identify NK cells. A stain using control IgG was used to calculate and remove background counts.

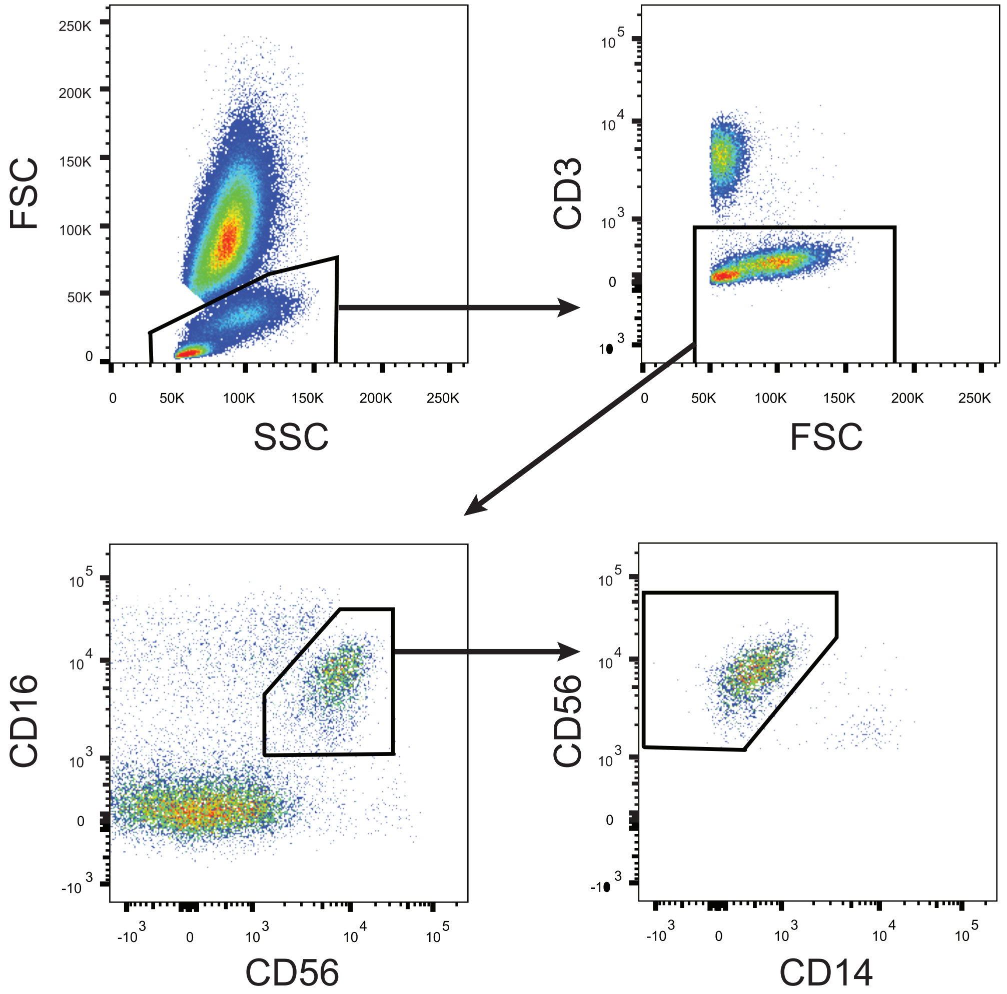


**Supplementary Figure 6.** Gating strategy for NK cells in peripheral blood in human participants. Panels of fluorescently labeled antibodies were used to identify immune cell populations via flow cytometry. Following elimination of doublets, NK cells were identified as side scatter-low, CD3-. Within this population, the NK cell population was CD56-mid, CD16+. CD14 staining was also used to eliminate monocyte contamination.

| **Supplemental Table 1.** NK cell surface markers | | | | | |
|---|---|---|---|---|---|
| **Marker** | **Function/Expression Pattern** | **MFI** | **Subgroups** | **Ref.** | **Antibody Catalogue #** |
| CCR4 | Trafficking; activation; subgroups | – | – | (1-3) | 359407 |
| CD11a | Cell adhesion; trafficking | + | – | (4) | 301207 |
| CD11b | Cell adhesion; trafficking; development | + | – | (4, 5) | 301323 |
| CD27 | Subgroups; inhibition receptor | - | + | (6, 7) | 124211 |
| CD38 | Activation receptor | + | – | (8, 9) | 356603 |
| CD40L | Cytotoxic activity | – | – | (10) | 310823 |
| CD45RA | Activation/activation history; development | + | – | (11, 12) | 304129 |
| CD57 | Maturation; NK cell subgroup | – | + | (13-15) | 322313 |
| CD62L | Maturation; polyfunctionality; migration | – | + | (16) | 304805 |
| CD69 | Activation | – | – | (17, 18) | 310929 |
| CD94 | Inhibition receptor | – | + | (18-20) | 305508 |
| CX3CR1 | Migration; adhesion | + | – | (3, 21-23) | 341603 |
| CXCR3 | Migration; adhesion | – | + | (24) | 353715 |
| FasL | Cytotoxic activity | – | – | (25) | 306406 |
| KIR2D L1/S1/S3/S5 | Activation receptor | – | + | (15, 26, 27) | 339505 |
| KIR2D L2/L3 | Activation receptor | – | + | (15, 26, 27) | 312611 |
| NKG2D | Cytotoxic activity | + | – | (5, 28-30) | 320821 |
| Nkp30 | Activation receptor | + | – | (31-33) | 325209 |
| Nkp44 | Activation receptor | – | – | (5, 34) | 325107 |
| Nkp46 | Activation receptor | + | – | (5, 33) | 331913 |
| TRAIL | Cytotoxic activity | – | – | (25) | 308209 |
| APC IgG | APC negative control | N/A | N/A | | 400120 |
| BV421 IgG | Brilliant Violet 421 negative control | N/A | N/A | | 306721 |
| PE IgG | PE negative control | N/A | N/A | | 400112 |
| N/A = not applicable | | | | | |

## References


1. Stolberg VR, Martin B, Mancuso P, Olszewski MA, Freeman CM, Curtis JL, et al. Role of CC chemokine receptor 4 in natural killer cell activation during acute cigarette smoke exposure. *The American journal of pathology.* 2014;184(2):454-63.
2. Inngjerdingen M, Damaj B, and Maghazachi AA. Human NK cells express CC chemokine receptors 4 and 8 and respond to thymus and activation-regulated chemokine, macrophage-derived chemokine, and I-309. *Journal of immunology.* 2000;164(8):4048-54.
3. Inngjerdingen M, Damaj B, and Maghazachi AA. Expression and regulation of chemokine receptors in human natural killer cells. *Blood.* 2001;97(2):367-75.
4. Ihanus E, Uotila L, Toivanen A, Stefanidakis M, Bailly P, Cartron JP, et al. Characterization of ICAM-4 binding to the I domains of the CD11a/CD18 and CD11b/CD18 leukocyte integrins. *European journal of biochemistry.* 2003;270(8):1710-23.
5. Freud AG, Yokohama A, Becknell B, Lee MT, Mao HC, Ferketich AK, et al. Evidence for discrete stages of human natural killer cell differentiation in vivo. *The Journal of experimental medicine.* 2006;203(4):1033-43.
6. Hayakawa Y, and Smyth MJ. CD27 dissects mature NK cells into two subsets with distinct responsiveness and migratory capacity. *Journal of immunology.* 2006;176(3):1517-24.
7. Silva A, Andrews DM, Brooks AG, Smyth MJ, and Hayakawa Y. Application of CD27 as a marker for distinguishing human NK cell subsets. *International immunology.* 2008;20(4):625-30.
8. Mallone R, Funaro A, Zubiaur M, Baj G, Ausiello CM, Tacchetti C, et al. Signaling through CD38 induces NK cell activation. *International immunology.* 2001;13(4):397-409.
9. Sconocchia G, Titus JA, Mazzoni A, Visintin A, Pericle F, Hicks SW, et al. CD38 triggers cytotoxic responses in activated human natural killer cells. *Blood.* 1999;94(11):3864-71.
10. Carbone E, Ruggiero G, Terrazzano G, Palomba C, Manzo C, Fontana S, et al. A new mechanism of NK cell cytotoxicity activation: the CD40-CD40 ligand interaction. *The Journal of experimental medicine.* 1997;185(12):2053-60.
11. Warren HS, and Skipsey LJ. Loss of activation-induced CD45RO with maintenance of CD45RA expression during prolonged culture of T cells and NK cells. *Immunology.* 1991;74(1):78-85.
12. Freud AG, Becknell B, Roychowdhury S, Mao HC, Ferketich AK, Nuovo GJ, et al. A human CD34(+) subset resides in lymph nodes and differentiates into CD56bright natural killer cells. *Immunity.* 2005;22(3):295-304.
13. Lopez-Verges S, Milush JM, Pandey S, York VA, Arakawa-Hoyt J, Pircher H, et al. CD57 defines a functionally distinct population of mature NK cells in the human CD56dimCD16+ NK-cell subset. *Blood.* 2010;116(19):3865-74.
14. Lopez-Verges S, Milush JM, Schwartz BS, Pando MJ, Jarjoura J, York VA, et al. Expansion of a unique CD57(+)NKG2Chi natural killer cell subset during acute human cytomegalovirus infection. *Proceedings of the National Academy of Sciences of the United States of America.* 2011;108(36):14725-32.
15. Bjorkstrom NK, Riese P, Heuts F, Andersson S, Fauriat C, Ivarsson MA, et al. Expression patterns of NKG2A, KIR, and CD57 define a process of CD56dim NK-cell differentiation uncoupled from NK-cell education. *Blood.* 2010;116(19):3853-64.
16. Juelke K, Killig M, Luetke-Eversloh M, Parente E, Gruen J, Morandi B, et al. CD62L expression identifies a unique subset of polyfunctional CD56dim NK cells. *Blood.* 2010;116(8):1299-307.

17. Clausen J, Vergeiner B, Enk M, Petzer AL, Gastl G, and Gunsilius E. Functional significance of the activation-associated receptors CD25 and CD69 on human NK-cells and NK-like T-cells. *Immunobiology.* 2003;207(2):85-93.
18. Borrego F, Robertson MJ, Ritz J, Pena J, and Solana R. CD69 is a stimulatory receptor for natural killer cell and its cytotoxic effect is blocked by CD94 inhibitory receptor. *Immunology.* 1999;97(1):159-65.
19. Bryceson YT, Ljunggren HG, and Long EO. Minimal requirement for induction of natural cytotoxicity and intersection of activation signals by inhibitory receptors. *Blood.* 2009;114(13):2657-66.
20. Posch PE, Borrego F, Brooks AG, and Coligan JE. HLA-E is the ligand for the natural killer cell CD94/NKG2 receptors. *Journal of biomedical science.* 1998;5(5):321-31.
21. Donnelly DJ, Longbrake EE, Shawler TM, Kigerl KA, Lai W, Tovar CA, et al. Deficient CX3CR1 signaling promotes recovery after mouse spinal cord injury by limiting the recruitment and activation of Ly6Clo/iNOS+ macrophages. *The Journal of neuroscience : the official journal of the Society for Neuroscience.* 2011;31(27):9910-22.
22. Imai T, Hieshima K, Haskell C, Baba M, Nagira M, Nishimura M, et al. Identification and molecular characterization of fractalkine receptor CX3CR1, which mediates both leukocyte migration and adhesion. *Cell.* 1997;91(4):521-30.
23. Huang D, Shi FD, Jung S, Pien GC, Wang J, Salazar-Mather TP, et al. The neuronal chemokine CX3CL1/fractalkine selectively recruits NK cells that modify experimental autoimmune encephalomyelitis within the central nervous system. *FASEB journal : official publication of the Federation of American Societies for Experimental Biology.* 2006;20(7):896-905.
24. Wendel M, Galani IE, Suri-Payer E, and Cerwenka A. Natural killer cell accumulation in tumors is dependent on IFN-gamma and CXCR3 ligands. *Cancer research.* 2008;68(20):8437-45.
25. Zamai L, Ahmad M, Bennett IM, Azzoni L, Alnemri ES, and Perussia B. Natural killer (NK) cell-mediated cytotoxicity: differential use of TRAIL and Fas ligand by immature and mature primary human NK cells. *The Journal of experimental medicine.* 1998;188(12):2375-80.
26. Fauriat C, Ivarsson MA, Ljunggren HG, Malmberg KJ, and Michaelsson J. Education of human natural killer cells by activating killer cell immunoglobulin-like receptors. *Blood.* 2010;115(6):1166-74.
27. Borrego F, Kabat J, Kim DK, Lieto L, Maasho K, Pena J, et al. Structure and function of major histocompatibility complex (MHC) class I specific receptors expressed on human natural killer (NK) cells. *Molecular immunology.* 2002;38(9):637-60.
28. Hayakawa Y, and Smyth MJ. NKG2D and cytotoxic effector function in tumor immune surveillance. *Seminars in immunology.* 2006;18(3):176-85.
29. Garofalo S, Cocozza G, Porzia A, Inghilleri M, Raspa M, Scavizzi F, et al. Natural killer cells modulate motor neuron-immune cell cross talk in models of Amyotrophic Lateral Sclerosis. *Nature communications.* 2020;11(1):1773.
30. Morgado S, Sanchez-Correa B, Casado JG, Duran E, Gayoso I, Labella F, et al. NK cell recognition and killing of melanoma cells is controlled by multiple activating receptor-ligand interactions. *Journal of innate immunity.* 2011;3(4):365-73.
31. Ferlazzo G, Tsang ML, Moretta L, Melioli G, Steinman RM, and Munz C. Human dendritic cells activate resting natural killer (NK) cells and are recognized via the NKp30 receptor by activated NK cells. *The Journal of experimental medicine.* 2002;195(3):343-51.
32. Pende D, Parolini S, Pessino A, Sivori S, Augugliaro R, Morelli L, et al. Identification and molecular characterization of NKp30, a novel triggering receptor involved in natural

cytotoxicity mediated by human natural killer cells. *The Journal of experimental medicine.* 1999;190(10):1505-16.

33. Campos C, Lopez N, Pera A, Gordillo JJ, Hassouneh F, Tarazona R, et al. Expression of NKp30, NKp46 and DNAM-1 activating receptors on resting and IL-2 activated NK cells from healthy donors according to CMV-serostatus and age. *Biogerontology.* 2015;16(5):671-83.
34. Marras F, Nicco E, Bozzano F, Di Biagio A, Dentone C, Pontali E, et al. Natural killer cells in HIV controller patients express an activated effector phenotype and do not up-regulate NKp44 on IL-2 stimulation. *Proceedings of the National Academy of Sciences of the United States of America.* 2013;110(29):11970-5.
